# Perspectives in the Treatment of *RAS* or *BRAF* Mutated Metastatic Colorectal Cancer Patients

**DOI:** 10.3389/fonc.2021.602596

**Published:** 2021-03-02

**Authors:** Gerardo Rosati, Giuseppe Aprile, Debora Basile, Antonio Avallone

**Affiliations:** ^1^Medical Oncology Unit, “S. Carlo” Hospital, Potenza, Italy; ^2^Medical Oncology Unit, “San Bortolo” Hospital, Vicenza, Italy; ^3^Department of Medical Oncology (DAME), University of Udine, Udine, Italy; ^4^Experimental Clinical Abdominal Oncology Unit, Istituto Nazionale Tumori- IRCCS-Fondazione G. Pascale, Napoli, Italy

**Keywords:** BRAF mutation, colorectal cancer, metastatic disease, RAS mutation, targeted therapies

## Background

Until a few years ago, the overall survival (OS) for metastatic colorectal cancer (mCRC) patients did not generally exceed 18–20 months in spite of a progressively evolving therapeutic armamentarium (1). Targeted therapies have revolutionized these results, leading to a marked increase in response rate (RR), progression-free survival (PFS), and OS. Despite these achievements, the benefits and toxicities may vary significantly from patient to patient and physicians’ decisions are best guided by the identification of predictive/prognostic biomarkers (2).

The development of a colorectal cancer, at least the sporadic ones (70–80%), proceeds over several years. Alongside an environmental genesis in which diet could be a cause, it is known that mutations in certain genes [neuroblastoma *RAS* (*NRAS*), Kirsten *RAS* (*KRAS*), mutant B rapidly accelerated fibrosarcoma (*BRAF*)], part of the downstream signaling pathways of the epidermal growth factor receptor (*EGFR*), are crucial in the process of carcinogenesis (3). Unfortunately, these mutations are also the main form of resistance to monoclonal antibodies such as cetuximab and panitumumab which, by binding to the extracellular domain of *EGFR*, inhibit cell growth which is otherwise nullified. The rat sarcoma virus (*RAS*) mutations of codons 12 and 13 of exon two, 59 and 61 of exon three, and 117 and 146 of exon four plus those of *BRAF* are therefore mandatory before treatment with anti-*EGFR* antibodies and can be found in about 60% of patients (4).

We here review the clinical usefulness of researching these mutations and express our opinion on the applicability and efficacy of potentially active compounds in these circumstances.

## Mutational Status of *RAS*

A pooled analysis of five randomized trials showed that mutant *KRAS* and *BRAF* (for *BRAF* see paragraph below) mCRC patients have shorter PFS and OS compared to those defined wild-type (wt), although not all the latter are sensitive to anti-*EGFR* antibodies (5). In these cases, *PIK3CA* gene mutation, phosphatase and tensin homolog (*PTEN*) loss, or human *EGFR*2 (*HER2*) amplification may lead to resistance to cetuximab and panitumumab. Mutated patients therefore need to be treated differently. While standard first-line chemotherapy doublets plus bevacizumab produce lesser benefits compared to wt patients, the combined use of oxaliplatin, irinotecan, and 5-fluorouracil (FOLFOXIRI) seems to bring better results (6). The data of second-line aflibercept and ramucirumab in combination with irinotecan and 5-fluorouracil (FOLFIRI) and those of regorafenib and trifluridine/tipiracil in pretreated patients suggest that these treatments are equally effective regardless of *RAS* mutations (7–9).

While uncommon *RAS* mutations—identified by more profound molecular analyses—might preclude the effectiveness of anti-*EGFR* antibodies in some wt patients, it is not clear how to eventually target *RAS*-mutant cancers. A meta-analysis showed that extending the *RAS* study to search for less frequent mutations has a significant impact on outcomes (10). A next-generation sequencing (NGS)-based companion diagnostic instrument has been validated to detect all *RAS* mutations in DNA extracted from formalin-fixed paraffin-embedded mCRC tumor samples (11). Although *RAS* is frequently mutated, its direct inhibition with specific target therapies (**Table 1**) has proved difficult.

**Table 1:** Preclinical and clinical trials for targeted therapies in RAS and BRAF metastatic CRC.

RAS- Preclinical studies		
Agent	Tumor types	Results
Anti-sense oligonucleotides AZD4785 (12)	KRAS mutant NSCLC, colon, pancreatic cell line xenografts and patient-derived xenografts	AZD4785 inhibited KRAS expression showing antitumor activity
Irinotecan and AZD2014 (15)	Four colon cancer cell lines Effects of treatments were tested on PDX	They significantly reduced ectopic patients-derived CRC tumor growth with more activity than FOLFOX or FOLFIRI. The combination inhibited also lung and liver localizations developed from orthotopic implantation of SW480 cells.
Temsirolimus and chloroquine (16)	SW480 and HT-29 CRC cells	The combination increased radiosensitivity in CRC cells through co-inhibition of mTOR and autophagy and strongly induced apoptosis in cells exposed to ionizing radiation
Combinations of MEK and PIK3CA inhibitors with BCL2L1 and/or BCL-2 inhibitors as double or triple regimens (17).	CRC lines	Combination increased antitumor activity overcoming resistant and aggressive cancer cells.
Combination of MEK inhibitors and anti-PD-L1 (21).	KRAS-mutant CRC	The combination of inhibition of MEK with PD-L1 resulted in a synergistic and durable tumor regression

RAS- Clinical trials						
Agent	N	Tumor types	Phase	Response	PFS	OS
Salirasib (13)	21	Advanced tumors	II	47.61% SD 42.85% PD	227 days	–
ARM A: Tipifarnib ARM B: Placebo (14)	368	Advanced CRC patients	III	–	ARM A: 81 days ARM B: 80 days	ARM A: 174 days ARM B: 185 days
Adagrasib (18)	17	*KRAS*^G12C^ mutant solid tumors	I/II	33.33% PR 66.66% SD	–	–
Sotorasib (19)	42	Advanced CRC patients	I	73.80% SD	4 months	–
Atezolizumab and cobimetinib (23)	84	Advanced CRC patients	I/Ib	8% PR 23% SD	1.9 months	9.8 months
ARM A: Atezolizumab and cobimetinib ARM B: Atezolizumab ARM C: Regorafenib (24)	363	Advanced CRC patients	III	–	–	ARM A:8.87 months ARM B: 7.10 months ARM C: 8.51 months
**BRAF- Clinical trials**						
**Agents**	**N**	**Tumor types**	**Phase**	**Response**	**PFS**	**OS**
ARM A: Encorafenib, cetuximab and binimetinib ARM B: Encorafenib and cetuximab ARM C: cetuximab and irinotecan or FOLFIRI BEACON CRC (33, 34)	665	Pretreated BRAF^V600E^ mutant mCRC patients	III	ARM A: 23% PR 42% SD ARM B: 15% PR 54% SD ARM C: 2% PR 29% SD	ARM A: 4.3 months ARM B: 4.2 months ARM C: 1.5 months	ARM A: 9.3 months ARM B: 9.3 months ARM C: 5.9 months
Encorafenib, binimetinib plus cetuximab, ANCHOR CRC (35)	41	Untreated BRAF ^V600E^ mutant mCRC patients	II	50% cORR	4.9 months	–

This is the case of anti-sense oligonucleotides that have been shown to induce genetic depletion of mutant *KRAS* in preclinical models (12). Salirasib is an oral *RAS* inhibitor that competitively blocks the membrane association of *RAS* proteins. Although with a reduced number of subjects, a Japanese phase I study showed that salirasib was safe and well tolerated, a dose of 800 mg twice daily was recommended for phase II studies, and patients showed PFS of 227 days (17).

Two farnesyltransferase inhibitors (FTI), tipifarnib and lonafarnib, were tested in phase III clinical trials in patients with advanced stage pancreatic cancer, NSCLC, mCRC, and acute myeloid leukemia. They showed no clinical efficacy in *KRAS*-driven cancer, leading to the conclusion that targeting post-translational modifications in *RAS* are ineffective (18).

Another way to achieve *RAS* inhibition may be to antagonize other proteins of its pathway, as in the case of mitogen-activated protein kinase (*MAPK*) or phosphatidylinositol 3-kinase (*PIK3CA*)/*Akt*/*mTOR* inhibitors. The *PI3K/Akt*/mammalian target of rapamycin (*mTOR*) plays an important role in intracellular functions, regulating cell proliferation, growth, cell size, metabolism and motility. Thus, the combination of irinotecan and AZD2014 significantly reduces cell invasion capacity by 70% and inhibits the growth of colon cancer derived from the ectopic patient, demonstrating greater potency than oxaliplatin and 5-fluorouracil (FOLFOX) or FOLFIRI. Furthermore, the combination totally inhibited the metastases developed by the orthotopic implantation of SW480 cells (13). Another study has shown that the combination of temsirolimus, *mTOR* inhibitor, and chloroquine, an autophagy inhibitor, increases radiosensitivity in colorectal cancer cells. In particular, ionizing radiation activated the proteins downstream of *mTOR* and induced autophagy, while chloroquine inhibited autophagy in the advanced stage and did not affect the proteins downstream of *mTOR* (14).

Another strategy is to use combined therapies. The anti-apoptotic protein BCL2L1 increases the efficacy of *MEK* inhibition, determining a strong cytostatic response and a further apoptotic action. Combinations of *MEK* and *PIK3CA* inhibitors with BCL2L1 and/or BCL-2 inhibitors as double or triple regimens showed increasing antitumor activity (15). This strategy mostly involves *MEK* inhibitors as a therapeutic backbone, although exploring combinations with newer agents that target other elements of the *MAPK* pathway could achieve a more optimal inhibition of *RAF-MEK-ERK* signaling. However, the limitation of these therapies is the tolerability that leads to a reduction in clinical activity through a reduction in doses.

More promising and in the process of clinical development are other molecules. Adagrasib binds to *KRAS*^G12C^, locking it in an inactive state. This inhibitor is currently being tested in a study involving 17 patients with *KRAS*^G12C^ mutant solid tumors, mostly non-small cell lung cancer or mCRC. Most of them reported low toxicity and 12 were valuable for the response: four had partial responses and eight had stable disease (SD). All responders received a dose of 600 mg twice a day; one with mCRC had a 47% reduction, 6 weeks after the start of treatment (19). Sotorasib, another small similar molecule, has been evaluated in 42 pretreated mCRC patients in a phase I study showing that at the 960 mg dose the most relevant grade 3 or 4 toxicities, diarrhea and anemia, occurred in 11.6% of cases, while 7.1% of patients had a confirmed response, and 31 of them (73.8%) had SD. The median progression-free survival was 4.0 months (range, 0.0+ to 11.1+) (20).

In addition, the use of immunotherapy may be effective in the presence of *RAS* mutations, since the reduction in the number of tumor-infiltrating lymphocytes (TILs), the negative influence on the tumor microenvironment, and the increase in the expression of programmed cell death ligand 1 (*PD-L1*) are often observed in *RAS*-mutated tumors. One study demonstrated an objective regression of lung metastases *via* a polyclonal CD8+ T cell response against mutant *KRAS* p.G12D in TILs obtained from a patient with mCRC (26). Preclinical evidence of *KRAS*-mutant colorectal cancer shows that targeted inhibition of mitogen-activated protein kinase (*MAPK*) (*MEK*) deeply blocks the naive priming of CD8 (+) T cells in tumor-bearing mice and increases their number inside. The combination of inhibition of *MEK* with *PD-L1* results in a synergistic and lasting tumor regression (16).

The combination of atezolizumab and the *MEK* inhibitor cobimetinib has also aroused interest. Although the efficacy of immunotherapy in patients with mCRC has generally been limited to tumors with mismatched DNA repair deficiencies or microsatellite instability (*MSI-H*) (27), responses were observed in 7 out of 84 patients (6 responders were microsatellite low/stable, 1 was microsatellite instable) in a phase I/Ib study (21). While the most common adverse events related to the treatment were diarrhea (67%), rash (48%), and fatigue (40%), clinical activity of atezolizumab and cobimetinib was reported regardless of *KRAS/BRAF* state. This potential synergistic activity was not confirmed in a subsequent phase III study, even though the recruitment of patients with *MSI-H* was no more than 5% emphasizing that the benefits of immunotherapy should be limited only to them (22).

## Mutational Status of *BRAF*

*BRAF*, a serine-threonine protein kinase, is the primary effector of *RAS* signaling and is mutated in roughly 10% of mCRC patients. *BRAF* mutations occur most frequently (>90%) at the valine 600 residue (BRAF^V600E^) and are usually and mutually exclusive with *RAS* mutations (28). The presence of the *BRAF*^V600E^ mutation characterizes a subgroup of patients with poor prognosis and modest benefit from standard treatments (29).

In recent years, FOLFOXIRI plus bevacizumab has been introduced as a possible standard of care in these cases (30). The use of this intensive combination was mainly supported by a subgroup analysis of 28 *BRAF*^V600E^ mutated patients in the TRIBE trial, which showed a median OS of 19.0 months in those treated with FOLFOXIRI/bevacizumab, whereas patients treated with FOLFIRI/bevacizumab had a shorter median OS of 10.7 months (31). However, TRIBE 2 trial did not confirm a greater benefit for *BRAF* mutant patients with FOLFOXIRI/bevacizumab compared to mFOLFOX6/bevacizumab (32). A potential major efficacy of an antiangiogenic agent in combination with chemotherapy has also been reported in second-line treatment of *BRAF*^V600E^ mCRC patients. A subgroup analysis of the VELOUR study showed that patients treated with aflibercept more FOLFIRI reported a doubled median OS than those treated with chemotherapy alone (33). However, the value of these data should be carefully considered given the small number of patients studied.

Unlike the favorable results observed in melanoma patients, treatment with *BRAF* inhibitor alone yielded low clinical activity in mCRC, due to insufficient inhibition of *MAPK* pathway through a feedback *EGFR* reactivation (34, 35). *BRAF* inhibitors were therefore combined with anti-*EGFR* antibodies plus or minus *MEK* inhibitors. A relevant clinical benefit of this strategy was demonstrated for the first time in the large, randomized phase III BEACON trial (23). In this trial, 665 *BRAF*^V600E^ mCRC patients, after one or two prior regimens, were randomized to receive the *BRAF* inhibitor encorafenib in combination with the anti-*EGFR* antibody cetuximab plus (triplet therapy) or minus the *MEK* inhibitor binimetinib (doublet therapy) or cetuximab in combination with irinotecan or FOLFIRI (standard therapy). The primary endpoints of the study were OS and RR. In the primary analysis, the triplet and doublet therapy, compared with standard therapy, resulted in significantly longer median OS (9.0, 8.4, and 5.4 months, respectively) and higher RR (26, 20, and 2%, respectively), with manageable safety profile. Interestingly, update results of the BEACON trial showed similar OS with either triplet and doublet combinations and a higher rate of adverse events in the first group compared to the other (65.8 *versus* 57.4%) (24). So, FDA approved encorafenib plus cetuximab in previously treated *BRAF*^V600E^ mCRC patients. This strategy has been foreshadowed in the first-line treatment of these patients. However, when examining the use of triplet therapy in the latter context, the first results of the phase II ANCHOR-CRC trial did not live up to expectations, reporting a median PFS of only 5 months (25).

Therefore, the role of the doublet and triplet inhibition of *EGFR* pathway in the first-line treatment of *BRAF*^V600E^ mCRC patients is still not determined and additional therapeutic strategies, addressing the rapid acquisition of resistance, are needed to improve the efficacy of *BRAF* inhibitors in this disease.

## Conclusions

While in recent years the possibility of overcoming the *BRAF* mutation has emerged more concretely, in our opinion *RAS* still remains a difficult target. Although the National Cancer Institute launched a program in 2013 to develop new promising agents through preclinical and clinical trials, their impact in the near future could be marginal for tumors harboring specific mutations, as they do not ensure a substantial change in therapeutic prospects for the majority of patients.

## Author Contributions

GR and AA wrote the manuscript. All authors participated in drafting and editing the manuscript. All authors contributed to the article and approved the submitted version.

## Funding

This work was supported by the Italian Ministry of Health (RF-2016-02363314).

## Conflict of Interest

The authors declare that the research was conducted in the absence of any commercial or financial relationships that could be construed as a potential conflict of interest.
